# Interleukin 1 receptor antagonist (*IL1RN*) gene variants predict radiographic severity of knee osteoarthritis and risk of incident disease

**DOI:** 10.1136/annrheumdis-2019-216055

**Published:** 2019-12-18

**Authors:** Mukundan Attur, Hua Zhou, Johathan Samuels, Svetlana Krasnokutsky, Michelle Yau, Jose U Scher, Michael Doherty, Anthony G Wilson, Jenny Bencardino, Marc Hochberg, Joanne M Jordan, Braxton Mitchell, Virginia B Kraus, Steven B Abramson

**Affiliations:** 1 Department of Medicine, Division of Rheumatology, New York University School of Medicine, New York, New York, USA; 2 Applied Bioinformatics Laboratories, New York University School of Medicine, New York, New York, USA; 3 Department of Epidemiology and Public Health, University of Maryland Baltimore, Baltimore, Maryland, USA; 4 Academic Rheumatology, University of Nottingham, Nottingham, UK; 5 University College Dublin, UCD School of Medicine and Medical Science, Conway Institute, Dublin, Ireland; 6 Radiology, New York University School of Medicine, New York, New York, USA; 7 Medicine/Epidemiology and Public Health, University of North Carolina, Chaple Hill, North Carolina, USA; 8 Departments of Medicine and Epidemiology, Thurston Arthritis Research Center, Chapel Hill, North Carolina, USA; 9 Geriatrics Research and Education Clinical Center, Baltimore VA Medical Center, Baltimore, Maryland, USA; 10 Medicine, Duke University Medical Center, Durham, North Carolina, USA

**Keywords:** osteoarthritis, interleukins, inflammation, genetics, rheumatoid arthritis

## Abstract

**Objective:**

In these studies, we examined the association of single nucleotide polymorphisms (SNPs) of the *IL1RN* gene with radiographic severity of symptomatic knee osteoarthritis (SKOA) and the risk of incident OA. We also explored these genetic polymorphisms in patients with new onset rheumatoid arthritis (RA).

**Methods:**

Over 1000 subjects who met American College of Rheumatology criteria for tibiofemoral OA were selected from three independent, National Institute of Health (NIH)-funded cohorts. CTA and TTG haplotypes formed from three SNPs of the *IL1RN* gene (rs419598, rs315952, rs9005) were assessed for association with radiographic severity, and risk for incident radiographic OA (rOA) in a nested case–control cohort. These *IL1RN* haplotypes were also assessed for association with disease activity (DAS28) and plasma inflammatory markers in patients with RA.

**Results:**

Carriage of the *IL1RN* TTG haplotype was associated with increased odds of more severe rOA compared with age-matched, sex-matched and body mass index-matched individuals. Examination of the osteoarthritis initiative *Incidence Subcohort* demonstrated that carriage of the TTG haplotype was associated with 4.1-fold (p=0.001) increased odds of incident rOA. Plasma IL-1Ra levels were lower in TTG carriers, while chondrocytes from TTG carriers exhibited decreased secretion of IL-1Ra. In patients with RA, the TTG haplotype was associated with increased DAS28, decreased plasma IL-1Ra and elevations of plasma inflammatory markers (hsCRP, interleukin 6 (IL-6)).

**Conclusion:**

Carriage of the *IL1RN* TTG haplotype is associated with more severe rOA, increased risk for incident OA, and increased evidence of inflammation in RA. These data suggest that the *IL1RN* TTG risk haplotype, associated with decreased IL-1Ra plasma levels, impairs endogenous ‘anti-inflammatory’ mechanisms.

**Key messages**

**What is already known about this subject?**

- Prior genetic studies have not identified any single causal locus with large effects on osteoarthritis (OA), but rather support the polygenic nature of the disease, consistent with the contribution of multiple variants with small effect sizes to variation in OA susceptibility or severity. The *IL-1* gene cluster region has been associated with susceptibility to OA in various joints, but the results have been inconsistent.

**What does this study add?**

- ➢The *IL1RN* associations that we describe in over 1000 patients with symptomatic knee OA are compelling because the risk haplotype is highly prevalent and has a large, biologically consistent effect on age-dependent radiographic severity or risk of incident disease.
- Our demonstration that the *IL1RN* risk haplotype is associated with more severe rheumatoid arthritis (RA) extends the biological implications to other chronic inflammatory conditions.
- From a pathogenic perspective, the association of the *IL1RN* TTG risk haplotype with decreased plasma IL-1Ra and increased IL-6/hsCRP suggests that carriers of the *IL1RN* TTG haplotype experience more severe and earlier disease due to genetically determined impaired ‘anti-inflammatory’ mechanisms.

**Key messages**

**How might this impact on clinical practice or future developments?**

- Drug development in OA would benefit from genetic biomarkers that identify individuals at greater risk for more severe or incident OA.
- Stratification by *IL1RN* risk haplotype in future clinical trial design and personalised medicine strategies could identify subsets of anti-IL1 responders/non-responders based on *IL1RN* risk haplotypes, as has been described in juvenile systemic arthritis.
- Finally, the understanding of the pathogenic mechanisms of *IL1RN* variants that impair effective endogenous anti-inflammatory mechanisms in OA and RA could lead to the identification of novel targets for treatment.

## Introduction

Osteoarthritis (OA) is characterised by focal loss of joint articular cartilage, osteophyte formation and subchondral bone remodelling. The production of interleukin 1 (IL-1β) and other mediators produced by cartilage and synovium induce a state of chronic low-grade inflammation that has been suggested to contribute to disease pathogenesis.^1–4^ Multiple genome-wide associations and candidate gene studies have identified genetic variants involved in the pathogenesis of OA,^5–9^ including variants in *ALDH1A2*, *GDF5*, *VDR*, *IGF-1*, *COL11A1* and *VEGF*. However, genetic studies have not identified any single causal locus, but rather are consistent with the contribution of multiple variants with small effect sizes to variation in OA susceptibility or severity.^10–12^

We have previously examined 15 single nucleotide polymorphisms (SNPs) in six inflammatory response genes, including those for *IL-1α, IL-1β, IL-1RN, TNFα, IL-10*, oestrogen receptor 1 (*ESR1*) and determined whether polymorphisms of these genes could predict risk for radiographic knee OA severity. We found that radiographic severity was associated only with a three SNP haplotype (rs419598, rs315952 and rs9005) of *IL1RN*, the product of which is IL-1Ra.^13^ The goal of this study was to validate these findings in over 1000 additional individuals with or at risk for knee OA and to determine whether the findings extended to patients with rheumatoid arthritis (RA).

## Methods

### Participants with symptomatic knee OA

We assembled 1066 subjects from three independent cohorts of individuals with or at risk for knee OA. Participants met clinical (American College of Rheumatology) and radiographic criteria for tibiofemoral OA (Kellgren-Lawrence (KL) score ≥1); all had body mass index (BMI) <33 kg/m^2^ (see online supplementary file 1). Using these eligibility criteria, we established a study population by including 300–400 subjects from each cohort with the goal of reducing phenotypic heterogeneity across populations. Radiographs were scored for tibiofemoral KL grade (0–4) and minimal medial joint space width (mJSW).^1 14–16^

10.1136/annrheumdis-2019-216055.supp1 Supplementary data

#### New York University OA cohort

To validate our original observation linking *IL1RN* haplotypes to OA severity from the ‘founding’ cohort of 80 New York University (NYU) and 50 Duke symptomatic knee osteoarthritis (SKOA) patients,^13 17^ we recruited and followed 372 additional SKOA patients between 2008 and 2016. Individuals who comprised the ‘founding’ cohort are not included in this study (NYUSoM IRB approved no: # i05-131 and i12-03682).

#### Genetics of Generalized Osteoarthritis

We applied the same inclusion/exclusion criteria to select a subset of participants in the Genetics of Generalized Osteoarthritis (GOGO) study from Duke University,^14^ and identified 339 individuals who met the eligibility criteria. None of the GOGO patients selected for this study were among the participants included in the previously reported ‘founding’ cohort.^13^

#### Osteoarthritis initiative

We applied identical criteria to select a subject subset from the osteoarthritis initiative (OAI), an observational cohort study focused on identifying genetic and clinical risk factors, imaging and biochemical biomarkers for development and progression of knee OA. The OAI study recruited individuals divided into two subcohorts, ‘Progression*’* and ‘Incidence*’*; inclusion and exclusion criteria for entry into the *Progression and Incidence Subcohorts* are available at http://oai.epi-ucsf.org/datarelease/.

#### Risk for SKOA

Using the OAI *Incidence Subcohort*, we performed a nested case–control study to assess the risk of incident disease. We identified 101 cases who developed either radiographic or symptomatic tibiofemoral radiographic knee OA within 2–4 years of baseline, and compared 101 controls who did not develop either frequent knee pain or radiographic tibiofemoral OA (>KL1) over a minimum of 4 years and for up to 96 months of follow-up, matched for gender, age and BMI at baseline visit (see online supplementary methods).

#### NYU new-onset RA cohort

All patients met the American College of Rheumatology/European League Against Rheumatism 2010 classification criteria for RA.^18^ Enrolled patients were seropositive: rheumatoid factor (95%); anti-citrullinated protein antibodies (100%). New-onset RA was defined as disease duration of a minimum of 6 weeks and up to 6 months since diagnosis, and absence of any treatment with disease-modifying anti-rheumatic drugs (DMARDs), biological therapy or steroids (ever) as we have described.^19^ Plasma samples from 145 RA subjects were selected for analysis. Clinical assessments included tender and swollen 28-joint counts, patient global disease activity assessment (0–100), and ESR to enable calculation of the DAS28-ESR.^20 21^

### Haplotype determination

Since all three SNPs (rs419598, rs315952 and rs9005) are in the *IL1RN* gene, we evaluated haplotype effects on radiographic severity as described in our previous publication.^13^ All cases and controls were genotyped for the same set of SNP markers (rs419598, rs315952 and rs9005) in the *IL1RN* gene. Of the nine potential haplotypes that could be constructed from these three SNPs, two occurred with a frequency that were >1% (haplotypes CTA and TTG). Both CTA and TTG are found on the same locus. Specifically, 61.7% of subjects could be unambiguously inferred to carry 0, 1, or 2 copies of the TTG haplotype, and 12% of subjects could be unambiguously inferred to carry 1 or 2 copies of the CTA haplotype. Throughout this report, we denote the TTG-0 or TTG-1 or TTG-2 haplotype groups, to represent carriers of 0, 1, or 2 copies of the IL1RN TTG haplotype generated from the 3 IL1RN SNPs (rs419598, rs315952 and rs9005). The linkage disequilibrium parameters D′ and r2 for IL1RN SNPs rs419598, rs315952 and rs9005 are shown in online supplementary table 1 for all three cohorts. In the GOGO cohort, rs9005 was not directly genotyped but was imputed with high quality (INFO >0.8). For consistency, we used the most probable imputed genotypes for all three SNPs to generate IL1RN haplotypes. For both rs315952 and rs419598 SNPs genotype concordance was excellent (r^2^=0.981 and 0.976, respectively).

10.1136/annrheumdis-2019-216055.supp2 Supplementary data

### Genetics and molecular analysis

Genotyping, cell culture assays and ELISA were performed as described in online supplementary file 1.

### Statistical analyses

Primary analyses evaluated associations between haplotypes and radiographic severity. Genotype associations with radiographic severity were determined using Fisher’s exact test, adjusted using the false discovery rate, where appropriate.

For a continuous trait outcome, mJSW versus age, we used a regression model. Age and mJSW correlation were plotted, and at each age interval, the likelihood of mJSW was calculated with a 95% CI (for additional information see online supplementary file 1).

## Results

### Frequency of IL1RN haplotypes

The clinical, genetic and demographic parameters in the three cohorts are shown in table 1. The frequencies of the *IL1RN* TTG haplotypes, based on SNPs rs419598, rs315952 and rs9005, were similar across the three cohorts. For the combined cohort of 1066 participants, the frequencies of TTG-0, TTG-1 and TTG-2 were 26.3%, 42.2% and 19.5%, respectively. The overall frequency of the CTA-1 or CTA-2 haplotype was 12.0% but varied across cohorts (NYU 7%; GOGO 20%; OAI 9%). Approximately 30% of the TTG-0 haplotype subjects in the combined cohort were CTA carriers.

**Table 1:** Demographic and radiographic characteristic features of symptomatic knee osteoarthritis patients from NYU, GOGO and OAI cohorts

Cohort	Age in years	Sex (% females)	BMI	Ethnicity (% Caucasians)	KL 3/4 (%)	mJSW in mm	Haplotype % frequencies				
							TTG-0	CTA-1/2 (TTG-0)	TTG-1 (CTA-0)	TTG-2	CTA-1 (TTG-1)
NYU (n=372)	61.4 (±10.4)	62.7	26.41 (±3.52)	64	48.9	3.21 (±1.53)	22.3	8.0	46.0	15.3	10.5
GOGO (n=339)	67.0 (±8.2)	75.5	26.8 (±3.40)	100	29.8	3.35 (±1.4)	32.0	20.0	41.0	27.0	0
OAI (n=355)	61.6 (±9.0)	56.9	29.95 (±4.90)	80	57.7	3.54 (±1.65)	24.2	9.3	40.0	17.2	16.4
Combined (n=1066)	62.7 (±9.9)	64.4	27.36 (±4.15)	68.5	43.2	3.24 (±1.61)	26.3	12.0	42.2	19.5	9.0

Details are shown of the mean (±SD) age, BMI and mJSW in millimetres (mm), as well as percentage of females, Caucasians, radiographic KL score 3 or 4 distribution and *IL1RN* TTG haplotype frequency distribution. The TTG-0 groups also include CTA-1/2 haplotype group. Haplotypes TTG-0 or TTG-1 or TTG-2, respectively, represent carriers of 0 or 1 or 2 copies of *IL1RN* haplotype produced using 3 *IL1RN* single nucleotide polymorphisms (rs419598, rs315952 and rs9005).

BMI, body mass index; GOGO, Genetics of Generalized Osteoarthritis study, Duke University; KL, Kellgren-Lawrence; mJSW, minimal medial joint space width; NYU, New York University School of Medicine; OAI, osteoarthritis initiative.

### *IL1RN* TTG haplotype is associated radiographic severity

**Figure 1:**
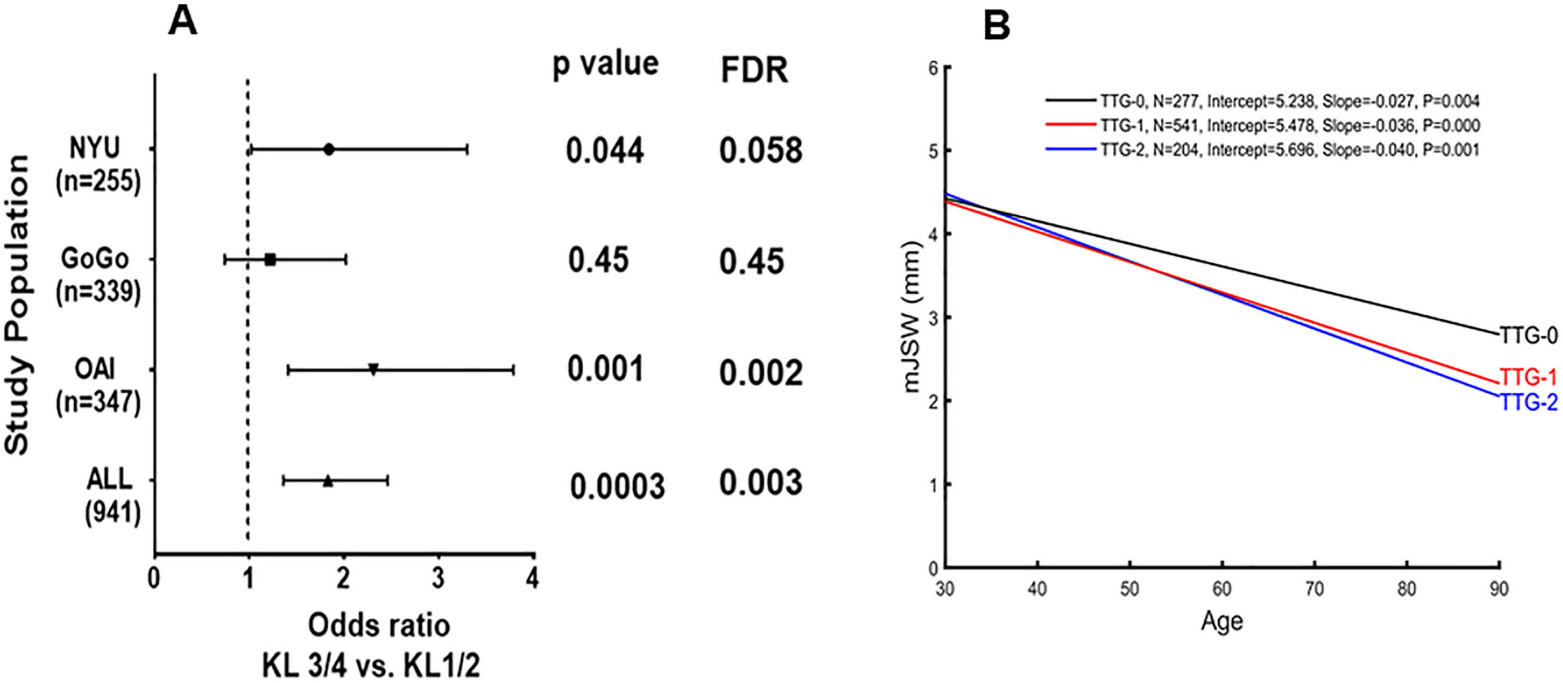
Association of *IL1RN* TTG haplotypes with radiographic severity. (A) Forest plot displaying association of *IL-1RN* haplotypes (TTG-0 vs TTG-1/2) with radiographic severity in symptomatic knee osteoarthritis (OA) patients in three cohorts. Study-specific estimates of ORs with 95% CIs between severity of knee OA defined as Kellgren-Lawrence (KL) 1/2 versus KL 3/4 for haplotype rs419598, rs315952 and rs9005 ‘T-T-G’ are shown for three independent and all three combined cohort. (B) Association of radiographic minimal medial joint space width (mJSW), age and *IL1RN* haplotypes in NYU, OAI and GOGO cohorts. Influence of IL-1 receptor antagonist (IL1RN) haplotypes on the age relationship to mJSW of knee OA. Carriers of either TTG-1 or TTG-2 compared with TTG-0 had narrower JSW (mm) at each age (years) studied. The joint space width (JSW) of each knee in patients with knee OA who do not (TTG-0) or do carry the *IL1RN* TTG haplotype is plotted relative to age, and the regression line is shown for JSW relative to age. The figure shows the linear regression line for each of the *IL1RN* risk haplotypes. OA patients 982 out of 1066 from three cohorts are represented. GOGO, Genetics of Generalized Osteoarthritis; FDR, false discovery rate; NYU, New York University; OAI, osteoarthritis initiative.

We first examined *IL1RN* TTG-0 versus TTG haplotypes (TTG-1 and TTG-2) for association with radiographic OA (rOA) severity as reported by KL scores and mean minimal medial radiographic joint space width (mJSW). As shown in a Forest plot (figure 1A), the *IL1RN* TTG haplotype was associated with an increased odds of more severe (KL 3/4 vs KL 1/2) radiographic knee OA compared with age-matched, sex-matched and BMI-matched knee OA patients with TTG-0 (OR of 1.83; 95% CI 1.36 to 2.46; p=0.0003). In the GOGO cohort, the TTG haplotype was associated with increased odds of KL 3/4 OA, which did not achieve significance. Of note, there was a lower percentage of participants with KL 3/4 OA severity (29.8%) in GOGO compared with the NYU (48.9%) and OAI (57.7%) cohorts, which may have reduced the statistical power of the test. We also note the higher frequency of the ‘protective’ CTA haplotype in the GOGO population (GOGO 20%; NYU 7%; OAI 9%), which could account for the smaller percentage of subjects with severe rOA, as we have reported.^8^

We next assessed radiographic severity by minimal medial joint space width (mJSW). Table 2 shows that relative to TTG-0, carriage of TTG-1 or TTG-2 was associated with a TTG ‘dose-dependent’ decrease in mJSW in each individual population. Linear regression analysis confirmed that compared with TTG-0, the TTG-1 or TTG-2 haplotypes were significantly associated with decreased mJSW (online supplementary table 2A). This TTG dose effect was also observed for KL severity (online supplementary table 2B). The risk haplotype TTG carriers did not associate with either Western Ontario and McMaster Universities Osteoarthritis Index (WOMAC) or Visual Analog Scale (VAS) pain in the NYU or OAI cohort.

**Table 2:** Association of IL1RN haplotype (TTG) with radiographic mean minimal medial joint space width (mJSW) in three combined (NYU, GOGO and OAI) cohorts of symptomatic knee osteoarthritis patients

Cohorts	TTG-0	TTG-1	TTG-2	Beta (95% CI)	P value	FDR
NYU (n=372)	3.43 (±1.44) (n=83)	3.28 (±1.45) (n=209)	2.60 (±1.70) (n=57)	−0.39 (−0.64 to −0.13)	0.0030	0.0046
GOGO (n=339)	3.67 (±1.31) (n=111)	3.08 (±1.51) (n=138)	3.35 (±1.38) (n=90)	−0.18 (−0.38 to 0.02)	0.0049	0.0950
OAI (n=355)	3.40 (±1.46) (n=86)	3.20 (±1.63) (n=200)	3.09 (±1.81) (n=61)	−0.16 (−0.42 to 0.10)	0.4787	0.2364
All (n=1066)	3.52 (±1.40) (n=280)	3.20 (±1.53) (n=547)	3.07 (±1.63) (n=208)	−0.23 (−0.37 to −0.10)	0.0023	0.0021
All meta-analysis (n=1066)	–	–	–	−0.23 (−0.37 to −0.10)	0.0008	0.0021

mJSW data are presented in millimetres as mean (±SD) unless otherwise indicated; number of subjects in each group is represented in square brackets below each value. Haplotypes TTG-0 or TTG-1 or TTG-2, respectively, represent carriers of 0 or 1 or 2 copies of *IL1RN* haplotype produced using 3 *IL1RN* single nucleotide polymorphisms (rs419598, rs315952 and rs9005). Linear regression model (beta and 95% CI) was performed and p value was adjusted by FDR. The last row indicates the meta-analysis of all three cohorts by including cohort as a covariate.

FDR, false discovery rate; GOGO, Genetics of Generalized Osteoarthritis; NYU, New York University; OAI, osteoarthritis initiative.

We next performed a more detailed analysis of the effects of different combinations of CTA and TTG haplotypes on mJSW. As shown in table 3, any combination of CTA-1 or CTA-2 was associated with wider mJSW compared with TTG-2 or TTG-1. For example, the mJSW for CTA-2 and CTA-1 or CTA-2 carriers was 3.67 (1.3) and 3.51 (1.31), respectively. In comparison, the mean mJSW for TTG-2 and TTG-1 or TTG-2 carriers was 3.07 (1.62) and 3.19 (1.57), respectively. The differences between CTA and TTG mJSW were significant after adjustment for common covariates age, sex and BMI (table 3).

**Table 3:** *IL1RN* TTG-1/2 carriers consistently associated with narrower joint space width in combined cohorts

	CTA-2	TTG-2	P value	P value_ ASB_ Adj	CTA-1/2 (TTG-0)	TTG-2	P value	P value_ ASB_ Adj	CTA-1/2 (TTG-0)	TTG-1/2 (CTA-0)	P value	P value_ ASB_ Adj
mJSW, mm	3.67 (±1.30)	3.07 (±1.62)	**0.0063**	**0.0072**	3.51 (±1.31)	3.07 (±1.62)	**0.0106**	**0.0124**	3.51 (±1.31)	3.19 (±1.57)	**0.0304**	**0.0123**
Age, years	65.01 (±8.24)	65.03 (±9.29)	0.9881	–	64.97 (±8.95)	65.03 (±9.29)	0.9498	–	64.97 (±8.95)	63.64 (±9.85)	0.1571	–
BMI	27.15 (±4.02)	27.74 (±3.98)	0.2933	–	27.31 (±3.66)	27.74 (±3.98)	0.3272	–	27.31 (±3.66)	27.55 (±4.20)	0.5478	–
Sex (% male)	36.76%	35.1%	–	–	33.6%	35.1%	–	–	33.6%	33.4%	–	–

Details are shown of the mean (±SD) age, BMI and mJSW in millimetres (mm), as well as percentage of males in each haplotype group in combined cohort (including New York University, Genetics of Generalized Osteoarthritis and osteoarthritis initiative). Haplotypes TTG-0 or TTG-1 or TTG-2, respectively, represent carriers of 0 or 1 or 2 copies of *IL1RN* haplotype produced using 3 *IL1RN* single nucleotide polymorphisms (rs419598, rs315952 and rs9005). Data are presented as mean (±SD) unless otherwise indicated. P values were determined by two-sample t-test and adjusted for ASB.

Bold indicates significant p values.

ASB, age, sex and BMI; BMI, body mass index; mJSW, minimal joint space width.

### *IL1RN* TTG haplotype predicts age-related rOA

We next evaluated the interaction between age, mJSW and *IL1RN* genotypes relative to radiographic severity. As shown in figure 1B, linear regression analysis demonstrated that carriers of either TTG-1 or TTG-2 compared with TTG-0 had narrower JSW (mm) at each age studied. At age 70, for example, mean mJSW was 3.34 mm in TTG-0 versus 2.86 mm in TTG-2 (p<0.005).

We analysed whether rOA was associated with *IL1RN* risk haplotype after adjustment for risk covariates (age, sex and BMI) in the regression model. As shown in online supplementary table 3, IL1RN risk haplotype carriers (TTG-1 or 2) had significantly narrower tibiofemoral mJSW compared with TTG-0 carriers; the association remained significant after adjustment for the covariates. In gender-specific analyses, we show that both male and female carriers of either TTG-1 or TTG-2 carriers had narrower mJSW compared with TTG-0 (online supplementary table 4). In addition, among Blacks and Hispanics TTG-1 or TTG-2 carriers had narrower mJSW compared with TTG-0 (online supplementary tables 5 and 6) with a p value of 0.05–0.10.

### *IL1RN* TTG haplotype predicts the risk of incident rOA

We examined participants from the *Incidence Subcohort* of the OAI, selecting the subgroup without clinical or radiographic evidence of knee OA at baseline. We identified 101 cases who developed either radiographic or symptomatic tibiofemoral radiographic knee OA within 2–4 years of baseline. Using a nested case–control approach, we selected 101 controls from the OAI *Incidence Subcohort* who did not develop either pain or radiographic tibiofemoral OA (>KL1) over a similar period matched for age, sex and BMI. These subjects were followed for 2–8 years. Table 4 shows that the presence of the *IL1RN* TTG-2 haplotype significantly increased the risk of incident knee rOA (OR=4.13 (1.75–9.72); p=0.001). After adjustment for age, sex and BMI with a logistic regression model, carriage of the TTG haplotype remained positively and significantly associated with incident rOA (beta coefficient=1.38; 95% CI 0.48 to 2.28; p=0.002).

**Table 4:** *IL1RN* TTG haplotype increases risk of incident osteoarthritis (OA)

	Age (years)	BMI	Sex	TTG-2	TTG-0	OR (95% CI); P value	Beta ASB adjusted
Cases	62.6±8.9	26.4±3.3	M=31; F=70	48 (M=14; F=34)	16 (M=7; F=9)	4.13 (1.75–9.72); **0.001**	1.38 (0.48–2.28); **0.002**
Controls	62.6±8.8	26.3±3.3	M=31; F=70	16 (M=6; F=10)	22 (M=0; F=22)		

Development of incident OA in cases was defined as development of frequent knee pain and radiographic OA (KL ≥1 or 2) in the same knee or in bilateral knees. Controls were individuals whose baseline Kellgren-Lawrence (KL)=0 or 1 did not change at follow-up AND who did not develop frequent pain in either knee at 24, 36, 48, 72 and 96 months. Cases and controls were matched for age, sex and BMI. Estimates of OR with 95% CIs between severity of knee OA defined as KL 1/2 versus KL 3/4 for haplotype rs419598, rs315952 and rs9005 ‘T-T-G’ are shown. Haplotypes TTG-0 or TTG-1 or TTG-2, respectively, represent carriers of 0 or 1 or 2 copies of *IL1RN* haplotype produced using 3 *IL1RN* SNPs (rs419598, rs315952 and rs9005). Data for age and BMI are presented as mean±SD; data for sex are presented as N of male and female. The ORs of patients falling into case or control groups versus *IL1RN* haplotype were calculated using Fisher’s exact test. Beta coefficient (and 95% CI) from logistic regressions were adjusted for ASB.

ASB, age, sex and BMI; BMI, body mass index.

### TTG risk haplotype is associated with decreased plasma IL-1Ra levels in patients with OA

Genetic variants of *IL1RN* have been associated with plasma levels of IL-1Ra and may regulate intracellular IL-1Ra protein trafficking.^22–25^ In our studies, mean plasma IL1Ra protein concentrations in TTG-2 carriers were lower than in CTA-2 carriers (346.50 vs 479.45 pg/mL, p=0.05, respectively). In this subset of patients, carriers of the CTA-2 haplotype had wider mean (SD) mJSW than did TTG-2 carriers (3.28 (1.46) vs 2.60 (1.67) mm, p=0.046, age-adjusted, sex-adjusted and BMI-adjusted). This was despite the fact that CTA-2 carriers were a mean 6 years *older* than the TTG-2 carriers (68.94 (9.92) vs 62.33 (10.96) years, p=0.08), consistent with an age-evident ‘protective’ effect of CTA on rOA.

We also performed a causal analysis to determine relationships among *IL1RN* TTG, CTA haplotypes, age, sex, BMI, IL-1Ra and mJSW. As shown in online supplementary figure 2, the CTA haplotype and BMI, but not age, are independently associated with plasma IL-1Ra. The causal analysis also indicated that the TTG haplotype directly associated with mJSW. As expected, both age and BMI associated with mJSW, and these effects were independent of the TTG haplotype.

10.1136/annrheumdis-2019-216055.supp3 Supplementary data

### *IL1RN* haplotypes in patients with RA

**Figure 2:**
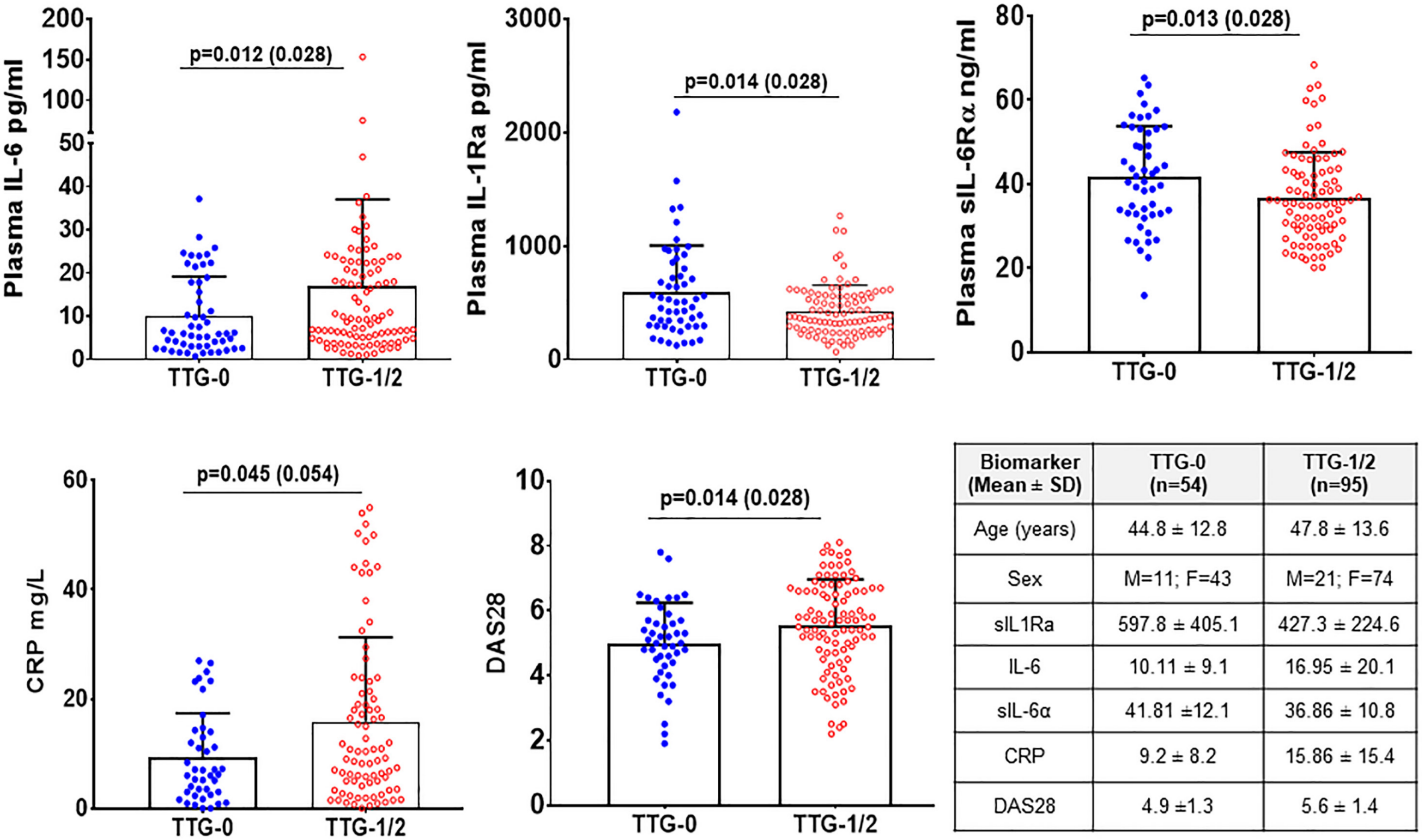
IL1RN TTG-risk haplotype carriers have decreased IL-1Ra, soluble IL-6Rα and increased IL-6, CRP accompanied by increased disease activity (DAS28) in rheumatoid arthritis patients. Plasma levels of biomarkers were determined using ELISA as described in methods. The mean (SD) age, sex and biomarkers and number of subjects in each haplotype group are shown in the table. Each dot represents individual sample. The solid horizontal bar in each group represents the mean and the vertical bar represents the positive SD values. Mann-Whitney U test was used to analyse significance difference between specific haplotype groups (TTG-1/2) with haplotype of TTG-0 groups. The p value and false discovery rate adjusted values (in brackets) are shown in the figures. CRP, C‐reactive protein.

We examined plasma samples from new-onset, DMARD-untreated patients with RA, followed at NYU.^20 21^ As shown in figure 2, carriers of the TTG risk haplotype exhibited lower levels of plasma IL-1Ra and the soluble IL-6 receptor antagonist alpha (sIL-6Rα) than age-matched, BMI-matched and sex-matched individuals with RA. Conversely, in TTG carriers plasma IL-6 and hsCRP were higher. Clinically, carriers of the TTG haplotype exhibited greater disease activity (DAS28).

### *IL1RN* haplotypes affect chondrocytes production of IL-1Ra

We next examined the relationship between TTG haplotypes and IL-1Ra production by chondrocytes. Chondrocytes were isolated from patients undergoing total joint replacement surgery at NYU, as described.^26^ Cell lysates and matched supernatants were analysed for IL-1Ra protein concentrations after 24 hours culture in the presence or absence of IL-1β. As shown in online supplementary table 7, following exposure to IL-β, basal levels of secreted IL-1Ra did not increase in TTG carriers, whereas *intracellular* concentrations of IL-1Ra in TTG chondrocytes were markedly increased. In contrast, chondrocytes obtained from TTG-0 individuals significantly increased the production of both intracellular and extracellular IL-1Ra following stimulation with IL-1β.

## Discussion

The *IL-1* gene cluster region has been associated with susceptibility to OA in various joints, but the results have been inconsistent.^27–32^ In this study of more than 1000 individuals with SKOA, we show that carriers of the *IL1RN* CTA haplotype (rs419598, rs315952 and rs9005) exhibit decreased age-dependent radiographic severity. Conversely, the TTG haplotype is associated with more severe rOA. Moreover, we demonstrate that the *IL1RN* TTG haplotype significantly increased the risk for incident tibiofemoral knee OA.

These results are consistent with our previous report that CTA in a large meta-analysis associated with less severe radiographic severity of knee OA.^17^ We note that in the genome-wide association study of OA using the UK Biobank, individual associations of each *IL1RN* SNP did not associate with knee OA at the genome-wide threshold (p<5×10^−8^).^33^ Similarly, in our study, *IL1RN* individual SNPs did not associate with knee OA (either KL or JSW).^17^ However, only the *IL1RN* haplotype, not tested in the UK Biobank study, was associated with more severe rOA in our studies. Another difference is the use of patient and/or hospital reported OA knee cases in the UK Biobank study, rather than radiographically confirmed SKOA as in our cohort, that may have resulted in a more heterogenous population of OA cases in the UK cohort.^6 33^

We also tested for association between the *IL1RN* haplotypes and radiographic progression, but neither of these associations were statistically significant. This is in contrast to the studies by Wu *et al*, who reported that the *IL1RN* TTG haplotype associated with change in KL over 4–11 years).^34^ Therefore, the lack of an association with progression in our studies could be a consequence of low power or insufficient years of follow-up. Alternatively, it is possible that exposures (eg, genotype) that increase disease susceptibility may also promote progression but such an association could be hard to detect because both progressors and non-progressors may already be enriched for the susceptibility genotype. This is a form of selection bias known as ‘collider bias’.

The association of *IL1RN* haplotypes with increased rOA at earlier age and the risk of incident disease may have clinical implications. Drug development in OA would benefit from genetic biomarkers that identify individuals at greater risk for more severe or incident OA.^35^ Stratification by *IL1RN* risk haplotype in future clinical trial design could identify subsets of anti-IL1 responders/non-responders based on *IL1RN* risk haplotypes, as has been described in juvenile systemic arthritis.^36^

What might be the biological explanation for the ‘*yin/yang’* genetic effects of CTA versus TTG on rOA? We have previously shown that individuals carrying the *IL1RN* CTA (TTG-0) haplotype had significantly lower synovial fluid levels of IL-10 and showed a trend towards lower levels of IL-1β and IL-6.^13^ We here report that in patients with both OA and RA, carriers of the TTG haplotype exhibit reduced plasma levels of IL-1Ra compared with CTA carriers. We provide evidence in chondrocytes that this may result from decreased secretion of IL-1Ra protein. Although our studies focused on cartilage, the source of IL-1Ra in the synovial joint fluid could be from various tissues in the joint, including inflamed synovium. We postulate that the greater severity of rOA in carriers of the TTG haplotype results from impaired antagonism of chronic inflammatory IL-1β-driven processes.^1 2^

In these studies, we also asked whether the association of the TTG haplotype with more severe disease was limited to OA, or could be demonstrated in patients with new onset RA. We found that in RA, as in OA, plasma levels of IL-1Ra were decreased in TTG carriers, and this was accompanied by *increased* plasma IL-6 and hsCRP in association with *increased* clinical disease activity (DAS28).

With regard to limitations, our studies were restricted to SKOA, since standardised radiographs of other joints were not available in each of the study cohorts. Therefore, the risk conferred by the *IL1RN* risk haplotypes to individuals with OA of the hip, hands, and/or the spine will need assessment in future studies. In addition, we note that participants enrolled in our three cohorts were predominantly North American Caucasian. However, although the numbers were small, subset analysis of Black and Hispanic subjects indicated a trend towards increased rOA severity in each subset (online supplementary tables 5 and 6). Thus, confirmation of these findings in Black, Asian and Hispanic populations will require future studies.

## Conclusion

In summary, we demonstrate that the *IL1RN* TTG haplotype identifies a subset of individuals with knee OA who are at increased risk for age-dependent rOA and increased risk for incident OA. Evidence for increased serological and clinical markers of disease activity in TTG carriers is also provided in new onset RA. We postulate that carriers of the *IL1RN* TTG haplotype experience more severe disease due to genetically determined impaired ‘anti-inflammatory’ mechanisms.
