# Supplementary material for: Interleukin 1 receptor antagonist (IL1RN) gene variants predict radiographic severity of knee osteoarthritis and risk of incident disease
Source: Ann Rheum Dis. 2019 Dec 18;79(3):400–7. doi: 10.1136/annrheumdis-2019-216055 (PMC7034355; doi:10.1136/annrheumdis-2019-216055)
Supplement: Supplementary data [file annrheumdis-2019-216055supp001.pdf]

SUPPLEMENTARY METHODS

Participants with symptomatic knee osteoarthritis (SKOA). In order to reduce phenotypic heterogeneity across the three study populations that would otherwise result from differences in enrollment criteria, demographics and study design, we applied comparable inclusion/exclusion criteria for identification of participants for the study in the NYU, GOGO and OAI *Progression Subcohort* populations. Inclusion criteria are noted in the main text. Exclusion criteria included histories of intra-articular corticosteroid injection within 3 months of enrollment, bilateral knee replacements, and other forms of arthritis, cancer or other chronic diseases beyond hypertension or hypercholesterolemia. Using the eligibility criteria described above, we assembled a study population by including 300-400 subjects from each of the three different cohorts identified below (NYU, GOGO, OAI).

Patients in all three cohorts underwent standardized weight-bearing fixed-flexion postero-anterior knee radiographs with a positioning frame to control the degree of flexion and leg rotation (SynaFlexer; Synarc, San Francisco, California, USA). Radiographs were scored for tibiofemoral Kellgren-Lawrence (KL) grade (0–4), and minimal joint space width (JSW) for each cohort as previously described.[1-4]

NYU cohort. To validate our original observation linking *IL1RN* haplotypes to OA severity from the "founding" cohort of 80 NYU and 50 Duke SKOA patients,[5, 6] we recruited and followed 372 additional SKOA patients between 2008-2016. Individuals who comprised the "founding" cohort are not included in this study.

Genetics of Generalized Osteoarthritis (GOGO). We used our NYU inclusion/exclusion criteria to select a subset of patients from the GOGO study from Duke University, designed to identify genetic susceptibility to generalized OA and OA progression.[3] Review of the GOGO cohort identified 339 patients who met the eligibility criteria and had *IL1RN* genotyping available for analysis. None of the selected GOGO cohorts were among the patients included in the previously reported “founding” cohort.[5]

Osteoarthritis Initiative (OAI). Similarly, we applied the NYU eligibility criteria to identify a corresponding subset from the OAI study; an observational cohort study focused on identifying genetic and clinical risk factors and imaging and biochemical biomarkers for development and progression of knee OA. A racially diverse group of 4,796 men and women between the ages of 45-79 years who had or were at risk for symptomatic knee OA were enrolled at 4 academic centers within the United States between February 2004 and May 2006. The OAI recruited groups of individuals divided into two sub-cohorts, those with symptomatic radiographic knee OA in at least one knee at risk of disease progression (the “*Progression*” sub-cohort), and those at high risk of initiation of symptomatic radiographic knee OA in one or both knees (the “*Incidence*” sub-cohort). Inclusion and exclusion criteria for entry into the *Progression and Incidence Sub-cohorts* are available at http://oai.epi-ucsf.org/datarelease/. Genomic DNA was obtained from the OAI Tissue Biorepository and x-ray image data from OAI. To assess radiographic severity, we selected 424 patients from the Progression sub cohort whose knee radiographs at baseline exhibited high-quality medial tibial plateau (MTP) alignment using OAI patient

identifiers provided by Dr. Eric Vignon, as described.[4] Of these, 355 patients met the NYU eligibility criteria for SKOA (age >35 years; BMI <33, and knee pain reported in the OAI database using the Numerical Rating Scale (NRS). Data used in the preparation of this article obtained from the Osteoarthritis Initiative (OAI) database, which is available for public access at http://www.oai.ucsf.edu/. Specific datasets used are [Version 25 release date 8/1/2017].

Selection and analysis of incident OA from the OAI. For genetic analyses of *IL1RN* haplotypes as predictors of the onset of incident OA, we analyzed the OAI *Incidence Subcohort.* We utilized a nested case-control design to study an equal number of age-, BMI-, co-morbidity- and sex-matched control patients who did not develop incident OA (Supplementary Figure 1 - Incidence flow chart).

At the time of enrollment in the *Incidence Subcohort,* subjects did not meet criteria for OA and were assessed at 24, 36, 48, 72 and 96 months. We focused on the subgroup with neither radiographic knee OA nor frequent knee pain at baseline, but with other risk factors for developing radiographic knee OA, as described (http://oai.epi-ucsf.org/datarelease/). Incident cases of symptomatic knee OA defined in the OAI as the first occurrence during the study of frequent knee symptoms in the presence of definite tibiofemoral knee OA (by OARSI grade 1-3) in the same knee. Analysis of the OAI database indicated that 792 participants had neither radiographic evidence of knee OA nor frequent knee pain at baseline, but had other risk factors for developing radiographic knee OA. We identified 101 OAI-defined incident cases diagnosed within 2-4 years of

baseline assessment based on two sub-group categories: ***a)*** symptomatic knee OA incidence; and ***b)*** radiographic-only incidence as shown in the chart. We then performed a nested case-controlled study, comparing 101 control subjects who did not meet criteria for incident OA over a minimum of 4 years and for up to 96 months of follow-up, matched for sex, age and BMI.

***IL1RN* genotyping for NYU, GOGO and OAI cohorts**. For the NYU cohort, genomic DNA from EDTA tubes was isolated using the QIAamp *DNA* Blood Mini *Kit* (Qiagen, USA). The DNA concentration estimated using Nanodrop. For the OAI cohort, we received blinded samples of purified genomic DNA from OAI biospecimen collection center. For the GOGO cohort, DNA was isolated as reported previously.[7] Genotyping for the *IL1RN* SNPs (rs419598 (C_8737990_10), rs315952 (C_11512470_10), rs9005 (C_3133528_10) for NYU and OAI cohorts were accomplished by polymerase chain reaction (PCR) using validated commercial SNP primers and probes (Applied Biosystems, CA, USA) along with detection using allelic discrimination computation (ABI Prism 7900HT Detection Systems). Genotypes were analyzed using TaqMan Genotyper Software version 1.4. All genotype and association studies were performed blinded to patient clinical data.

Briefly, all GOGO participants with longitudinal data were genotyped at the David H. Murdock Research Institute (Kannapolis, NC) using the Illumina BeadChip that consisted of 550,000 HapMap SNPs and 60,000 custom SNPs. Genotypes were called with Illumina GenomeStudio. Samples with call rates less than 95%, sex-discordance, excess

heterozygosity, or cryptic relatedness were removed (12 samples), leaving 1,245 samples for analysis. We removed SNPs with low call rates less than 99%, deviation from Hardy-Weinberg equilibrium ($p < 1\times10^{-3}$), and minor allele frequencies less than 5%, resulting in 478,118 genotyped SNPs that passed quality control. We additionally removed 1,110 ambiguous SNPs (with A/T or C/G alleles) and 593 SNPs that did not map to Genome Reference Consortium Human Build 37. A total of 478,118 SNPs were used as input for phasing and genotype imputation to the 1000 Genomes Phase I integrated haplotypes reference panel (December 2013) using SHAPEIT and IMPUTE2.[8, 9] All three SNPs analyzed – rs9005, rs315952 and rs419598 – were imputed with high quality (INFO > 0.8), of which the latter two SNPs were also directly genotyped. The imputed genotypes for two of the three SNPs in the haplotype (rs315952 and rs419598) were well-matched to the direct genotypes. These directly typed SNPs would also contribute information needed to obtain a good estimate of the imputed genotype for the third SNP (rs9005), For consistency, we used the imputed genotypes for all three SNPs in these analyses. In the absence of available direct genotyping, imputation is a valid tool for obtaining information on SNPs that were not directly genotyped[10].

Blood sample collection. For the NYU cohort, non-fasting blood samples were collected at baseline in pyrogen-free heparinized or EDTA tubes for isolation of plasma and genomic DNA, respectively. Plasma samples were immediately aliquoted and stored at -80°C for future use.

Chondrocyte functional studies. OA cartilage obtained from 45 separate patients undergoing total knee replacement surgery at NYU Langone Orthopedic Hospital and chondrocytes were isolated as described previously.[11, 12] Chondrocytes are were grown at a density of 5 x 10^5 cells/ well in six-well plates. The cells were adapted to serum-free media overnight and stimulated with IL-1β (10ng/ml) for 24h. Total protein extracted as described previously[11, 12] and supernatants collected for determination of inflammatory mediators. *IL1RN* genotyping performed as described above using TaqMan allelic discrimination assay.

IL-1Ra ELISA assay. Plasma, chondrocyte culture supernatants, and total cell lysates analyzed for IL-1Ra levels using a Human IL-1Ra/IL-1F3 Quantikine ELISA Kit (DRA00B from R&D systems) as described previously.[2] The intra- and inter-assay coefficients of variation for IL-1Ra measurements were 2.9% and 4.7%, respectively. Measurable values (>lower limit of detection, LLOD) were obtained for all samples assessed, and therefore, no imputation was necessary for statistical analyses.

Statistical analyses. *IL1RN* haplotype frequencies were calculated in SKOA patients in all three cohorts. Three *IL1RN* SNPs, previously implicated in risk of knee OA, rs419598, rs315952, and rs9005 were tested for association with knee OA severity. Haplotypes TTG-0 or TTG-1 or TTG-2 represent carriers of either 0 or 1 or 2 copies of *IL1RN* haplotype produced using 3 *IL1RN* SNPs (rs419598, rs315952 and rs9005). We excluded subjects with CTA/TTG haplotype heterozygotes from the analysis. Primary analyses evaluated associations between haplotypes and radiographic severity, as measured by

KL scores. To be consistent with our previously published work we classified patients as severe knee OA if the K/L score was 3 or 4 and as mild to moderate knee OA with a K/L score of 1 or 2. Genotype associations with radiographic severity were determined using Fisher's exact test, adjusted using false rate discovery (FDR), where appropriate, we also performed linear regression conditioning out effects of age/sex/ body mass index (BMI) by adding age/sex/BMI as covariates.

For a continuous trait outcome, i.e., mJSW v. age, a regression model was used. Age and mJSW correlation were plotted, and at each age interval, the expectation of mJSW was calculated with a 95% confidence interval. The difference of mJSW for each haplotype group within each age interval was evaluated using Student's t-test. For each cohort, we also fitted a linear regression of the *IL1RN* haplotypes against mJSW, beta coefficients, and p-values for *IL1RN* haplotypes were reported (Supplementary Tables). For this process, we also performed separate modeling after conditioning out effects of age/sex/BMI/ by adding age/sex/BMI/cohort as covariates.

Ethics approval and consent to participate. The current study was performed in accordance with the ethical standards of the Helsinki Declaration of 1975, as revised in 2000, and studies (#i9018 and i12-03682) were approved by the Institutional Review Board (IRB) of NYU School of Medicine. All patients provided written, informed consent to participate in the study.

REFERENCES FOR SUPPLEMENTARY METHODS

1. Attur M, Krasnokutsky S, Statnikov A, et al. Low-grade inflammation in symptomatic knee osteoarthritis: prognostic value of inflammatory plasma lipids and peripheral blood leukocyte biomarkers. Arthritis Rheumatol. 2015;67(11):2905-15.
2. Attur M, Statnikov A, Samuels J, et al. Plasma levels of interleukin-1 receptor antagonist (IL1Ra) predict radiographic progression of symptomatic knee osteoarthritis. Osteoarthritis Cartilage. 2015;23(11):1915-24.
3. Kraus VB, Jordan JM, Doherty M, et al. The Genetics of Generalized Osteoarthritis (GOGO) study: study design and evaluation of osteoarthritis phenotypes. Osteoarthritis Cartilage. 2007;15(2):120-7.
4. Mercier C, Piperno M, Vignon E, Brandt K, Hochberg M, Hellio Le Graverand MP. In normal knees, joint space width (JSW) is correlated with the intermargin distance (IMD), a measure of medial tibial plateau alignment. Variations in IMD explain variability in JSW in serial radiographs. Joint Bone Spine. 2013;80(2):183-7.
5. Attur M, Wang HY, Kraus VB, et al. Radiographic severity of knee osteoarthritis is conditional on interleukin 1 receptor antagonist gene variations. Ann Rheum Dis. 2010;69(5):856-61.
6. Kerkhof HJ, Doherty M, Arden NK, et al. Large-scale meta-analysis of interleukin-1 beta and interleukin-1 receptor antagonist polymorphisms on risk of radiographic hip and knee osteoarthritis and severity of knee osteoarthritis. Osteoarthritis Cartilage. 2011;19(3):265-71.
7. Castano-Betancourt MC, Evans DS, Ramos YF, et al. Novel Genetic Variants for Cartilage Thickness and Hip Osteoarthritis. PLoS Genet. 2016;12(10):e1006260.
8. Delaneau O, Marchini J, Zagury JF. A linear complexity phasing method for thousands of genomes. Nat Methods. 2011;9(2):179-81.
9. Howie BN, Donnelly P, Marchini J. A flexible and accurate genotype imputation method for the next generation of genome-wide association studies. PLoS Genet. 2009;5(6):e1000529.
10. Marchini J, Howie B. Genotype imputation for genome-wide association studies. Nat Rev Genet. 2010;11(7):499-511.
11. Attur M, Yang Q, Shimada K, et al. Elevated expression of periostin in human osteoarthritic cartilage and its potential role in matrix degradation via matrix metalloproteinase-13. FASEB J. 2015;29(10):4107-21.
12. Katsara O, Attur M, Ruoff R, Abramson SB, Kolupaeva V. Increased Activity of the Chondrocyte Translational Apparatus Accompanies Osteoarthritic Changes in Human and Rodent Knee Cartilage. Arthritis Rheumatol. 2017;69(3):586-97.
