# Supplementary material for: Interleukin 1 receptor antagonist (IL1RN) gene variants predict radiographic severity of knee osteoarthritis and risk of incident disease
Source: Ann Rheum Dis. 2019 Dec 18;79(3):400–7. doi: 10.1136/annrheumdis-2019-216055 (PMC7034355; doi:10.1136/annrheumdis-2019-216055)
Supplement: Supplementary data [file annrheumdis-2019-216055supp002.pdf]

***Supplementary Table* 1.** Linkage disequilibrium parameters D′ and r2 for *IL1RN* single nucleotide polymorphisms (SNPs) rs419598, rs315952 and rs9005 for all three cohorts: New York University School of Medicine (NYU), Genetics of Generalized Osteoarthritis (GOGO) and Osteoarthritis Initiative (OAI).

**SNP pair**	**D' / r^2**		
	**NYU**	**GOGO**	**OAI**
rs419598 / rs315952	0.62 / 0.21	0.72 / 0.22	0.57 / 0.22
rs419598 / rs9005	0.81 / 0.61	0.86 / 0.75	0.72 / 0.67
rs35952 / rs9005	0.68 / 0.31	0.99 / 0.35	0.82 / 0.35

Supplementary Table 2: IL1RN **haplotype TTG association with mJSW and radiographic severity in combined (NYU, GOGO, and OAI) cohorts.**

**Panel A. *IL1RN* SNP All Combined (n=1066)**	**mJSW: beta (± SE), p value, FDR**
**TTG-0** vs. TTG-1 haplotype	-0.35 (0.11), p=0.001, FDR=0.004
**TTG-0** vs. TTG-2 haplotype	-0.45 (0.14), p=0.001, FDR=0.007
**Panel B: *IL1RN* SNP All Combined (n=1066)**	**KL severity: OR (95% CI), p value, FDR**
**TTG-0** vs. TTG-1 haplotype	1.53 (1.12-2.08), p=0.008, FDR=0.017
**TTG-0** vs. TTG-2 haplotype	1.69 (1.27-2.24), p=0.000, FDR=0.001

In panel A, an association of medial joint space width (mJSW) in millimeters (mm) with IL1RN haplotypes were studied by comparing the indicated haplotype (TTG-0) with TTG-1 or TTG-2 using linear regression analysis. In panel B, Patients were classified by radiographic severity of knee OA by stratifying the KL scores to compare KL 1 or 2 versus KL 3 or 4. Odds ratios (OR) of KL vs. IL1RN haplotype also shows the association of Kellgren-Lawrence (KL) score with IL1RN haplotype.

Supplementary Table 3: **Regression analysis of *IL1RN* haplotype (TTG) with radiographic medial joint space width (mJSW) in three cohorts of symptomatic knee OA patients.**

	**ALL (n=1022)**<br>**Beta (SE); P=value**
**mJSW (mm) (Baseline), TTG Haplotype (0 vs. 1/2)**	
crude	-0.35 (0.11); 0.001
adjusted for age, sex	-0.35 (0.10); 0.001
adjusted for age, sex, BMI	-0.36 (0.10); 0.001
**mJSW (mm) (Baseline), TTG Haplotype (0 vs 2)**	
crude	-0.45 (0.14); 0.001
adjusted for age, sex	-0.40 (0.14); 0.004
adjusted for age, sex, BMI	-0.40 (0.14); 0.003

Medial joint space width (mJSW) in millimeters comparing the indicated haplotype (TTG-0) with TTG-1 or TTG-2. Haplotypes TTG-0 or TTG-1 or TTG-2, respectively, represent carriers of 0 or 1 or 2 copies of *IL1RN* haplotype produced using 3 *IL1RN* SNPs (rs419598, rs315952 and rs9005). Linear regression was performed unadjusted, adjusted for age and sex, as well as adjusted for age, sex, and body mass index (BMI) together.

Supplementary Table 4: **Association of *IL1RN* haplotype (TTG) in male and female with radiographic signal knee medial joint space width (mJSW) in combined (NYU, GOGO, and OAI) cohorts of symptomatic knee OA patients.**

	**Beta (SE), p-value**
**mJSW (mm) (Baseline), TTG Haplotype (0 vs 1/2) – Male**	**ALL (n=355)**
Crude	-0.48 (0.20), 0.018
adjusted for age, sex	-0.54 (0.19), 0.006
adjusted for age, sex, BMI	-0.50 (0.19), 0.009
**mJSW (mm) (Baseline), TTG Haplotype (0 vs 2) – Male**	**ALL (n=164)**
Crude	-0.58 (0.26), 0.027
adjusted for age, sex	-0.51 (0.25), 0.042
adjusted for age, sex, BMI	-0.49 (0.24), 0.047
**mJSW (mm) (Baseline), TTG Haplotype (0 vs 1/2) – Female**	**ALL (n=667)**
Crude	-0.28 (0.12), 0.021
adjusted for age, sex	-0.27 (0.12), 0.025
adjusted for age, sex, BMI	-0.28 (0.12), 0.023
**mJSW (mm) (Baseline), TTG Haplotype (0 vs 2) – Female**	**ALL (n=317)**
Crude	-0.38 (0.16), 0.018
adjusted for age, sex	-0.34 (0.16), 0.033
adjusted for age, sex, BMI	-0.34 (0.16), 0.034

Medial joint space width (mJSW) in millimeters comparing the indicated haplotype (TTG-0) with TTG-1 or TTG-2. Haplotypes TTG-0 or TTG-1 or TTG-2, respectively, represent carriers of 0 or 1 or 2 copies of *IL1RN* haplotype produced using 3 *IL1RN* SNPs (rs419598, rs315952 and rs9005). A linear regression model was performed and adjusted for age and sex, as well as for age, sex, and body mass index (BMI) in the combined cohort.

Supplementary Table 5: **Association of *IL1RN* haplotype (TTG) in Blacks with radiographic signal knee medial joint space width (mJSW) in combined (NYU and OAI) cohorts of symptomatic knee OA patients.**

	**Beta (SE), p-value**
**mJSW (mm) (Baseline), TTG Haplotype (0 vs 1/2)**	**ALL (n=82)**
Crude	-0.48 (0.34), 0.165
adjusted for age, sex	-0.46 (0.34), 0.183
adjusted for age, sex, BMI	-0.44 (0.34), 0.201
**mJSW (mm) (Baseline), TTG Haplotype (0 vs 2)**	**ALL (n=45)**
Crude	-0.77 (0.44), 0.091
adjusted for age, sex	-0.79 (0.44), 0.080
adjusted for age, sex, BMI	-0.82 (0.45), 0.072

Medial joint space width (mJSW) in millimeters comparing the indicated haplotype (TTG-0) with TTG-1 or TTG-2. Haplotypes TTG-0 or TTG-1 or TTG-2, respectively, represent carriers of 0 or 1 or 2 copies of *IL1RN* haplotype produced using 3 *IL1RN* SNPs (rs419598, rs315952 and rs9005). A linear regression model was performed and adjusted for age and sex, as well as for age, sex, and body mass index (BMI), in the combined cohort.

Supplementary Table 6: **Association of *IL1RN* haplotype (TTG) in Hispanic and non-Hispanic with baseline radiographic signal knee medial joint space width (mJSW) in combined (NYU and OAI) cohorts of symptomatic knee OA patients.**

**Hispanic**	**Beta (SE); P value**
mJSW (mm), TTG Haplotype (0 vs 1/2)	ALL (n=347)
crude	-0.39 (0.24); 0.106
adjusted for age, sex	-0.33 (0.24); 0.160
adjusted for age, sex, BMI	-0.38 (0.24); 0.108
mJSW (mm), TTG Haplotype (0 vs 2)	ALL (n=122)
crude	-0.56 (0.29); 0.052
adjusted for age, sex	-0.37 (0.27); 0.176
adjusted for age, sex, BMI	-0.42 (0.27); 0.126
**Non-Hispanic**	**Beta (SE); P value**
mJSW (mm), TTG Haplotype (0 vs 1/2)	ALL (n=587)
crude	-0.33 (0.17); 0.057
adjusted for age, sex	-0.29 (0.17); 0.088
adjusted for age, sex, BMI	-0.31 (0.17); 0.066
mJSW (mm), TTG Haplotype (0 vs 2)	ALL (n=211)
crude	-0.63 (0.22); 0.005
adjusted for age, sex	-0.49 (0.21); 0.022
adjusted for age, sex, BMI	-0.49 (0.21); 0.021

Medial joint space width (mJSW) in millimeters comparing the indicated haplotype (TTG-0) with TTG-1 or TTG-2. Haplotypes TTG-0 or TTG-1 or TTG-2, respectively, represent carriers of 0 or 1 or 2 copies of *IL1RN* haplotype produced using 3 *IL1RN* SNPs (rs419598, rs315952 and rs9005). A linear regression model was performed and adjusted for age and sex, as well as for age, sex, and body mass index (BMI), in the combined cohort.

Supplementary Table 7: **The *IL1RN* risk haplotype TTG (1 or 2) associated with decreased soluble IL-1Ra levels in chondrocytes**

	**Unstimulated, mean (±SD)**			**IL-1 induced, mean (±SD)**		
	**Extra-cellular (s)**	**Intra-cellular (ic)**	**Ratio (s vs. ic)**	**Extra-cellular (s)**	**Intra-cellular (ic)**	**Ratio (s vs. ic)**
***IL1RN* haplotype**						
TTG-1,2 (n=15)	5.05 (±8.1)	7.93 (±14.9)	1 : 1.2	6.19 (±7.1)	33.71 (±22.0)	1 : 5.4
TTG-0 (n=14)	1.46 (±2.2)	1.41 (±2.5)	1 : 1	10.36 (±11.9)	12.28 (±8.4)	1 : 1.2
**P-value**						
TTG-1, 2 vs. TTG-0	**0.05**	0.06	—	0.22	0.07	—
TTG-1,2 – Con vs. IL-1	—	—	—	0.62	**0.04**	—
TTG-0 – Con vs. IL-1	—	—	—	**0.01**	**0.0005**	—

We analyzed total cell lysate for extracellular and cell-associated IL-1Ra protein expression in primary chondrocytes by ELISA. Haplotypes TTG-0 or TTG-1 or TTG-2, respectively, represent carriers of 0 or 1 or 2 copies of *IL1RN* haplotype produced using 3 *IL1RN* SNPs (rs419598, rs315952 and rs9005). Chondrocytes with *IL1RN* TTG-1/2 haplotype produced significantly lower levels of soluble IL1Ra than TTG-0 carriers in both spontaneous conditions and following 24h IL-1β induction. The data represent mean (±SD), for n=15 individuals with TTG-1/2 and n=14 individuals with TTG-0. Mann Whitney U test was used to analyze difference between groups.
