# Supplementary material for: Interleukin 1 receptor antagonist (IL1RN) gene variants predict radiographic severity of knee osteoarthritis and risk of incident disease
Source: Ann Rheum Dis. 2019 Dec 18;79(3):400–7. doi: 10.1136/annrheumdis-2019-216055 (PMC7034355; doi:10.1136/annrheumdis-2019-216055)
Supplement: Supplementary data [file annrheumdis-2019-216055supp003.pdf]

***Supplementary Figure 1:*** **Incidence flow chart – selection and analysis of incident OA from the OAI.**

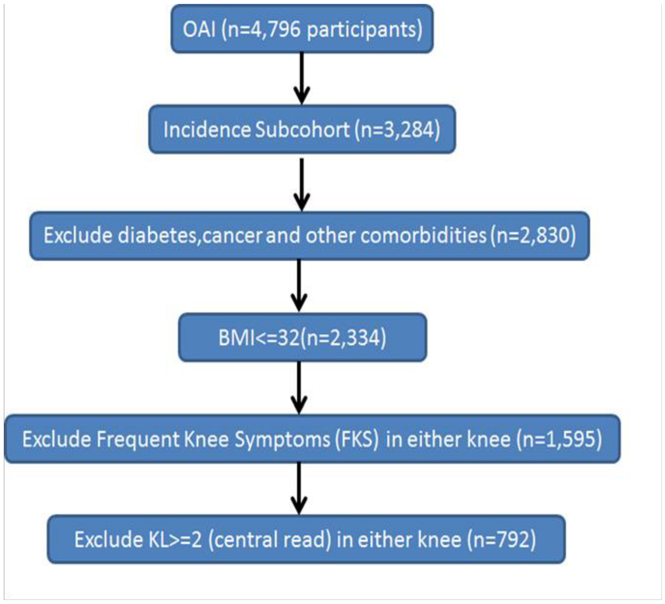


**Age, Gender, BMI-matched Controls (n=101)**

Baseline KL= 0 or 1 without change at follow up **AND** no FKS in either knee @24, 36, 48, 72, 96 mo.

**Cases (n=101 total)**

a) Development of FKS and Radiographic OA (KL ≥ 2) in the same knee or in bilateral knees (n=24)
b) Development of FKS and KL=1 when starting with KL=0 (n=21)
c) Development of radiographic incidence only (KL ≥ 2), (n= 56)

For genetic analyses of *IL1RN* haplotypes as predictors of the onset of incident OA, we analyzed the OAI *Incidence Subcohort.* We utilized a nested case-control design to study an equal number of age-, BMI-, comorbidity- and sex-matched control patients who did not develop incident OA. At the time of enrollment in the *Incidence Subcohort,* subjects did not meet criteria for OA and were assessed at 24, 36, 48, 72, and 96 months. We focused on the subgroup with neither radiographic knee OA nor frequent knee pain at baseline, but with other risk factors for developing radiographic knee OA, as described (http://oai.epi-ucsf.org/datarelease/). Incident cases of symptomatic knee OA are defined in the OAI as the first occurrence during the study of frequent knee symptoms in the presence of definite tibiofemoral knee OA (by OARSI grade 1-3) in the same knee. Analysis of the OAI database indicated that 792 participants had neither radiographic evidence of knee OA nor frequent knee pain at baseline, but had other risk factors for developing radiographic knee OA. We identified 101 OAI-defined incident cases diagnosed within 2-4 years of baseline assessment based on two subgroup categories: ***a)*** symptomatic knee OA incidence and ***b)*** radiographic-only incidence, as shown in the chart. We then performed a nested case-controlled study, comparing 101 control subjects who did not meet criteria for incident OA over a minimum of 4 years and for up to 96 months of follow-up, matched for sex, age, and BMI.

***Supplementary Figure 2:*** **Causal analysis of baseline biomarkers along with age, sex, and BMI on medial joint space narrowing (JSN).**

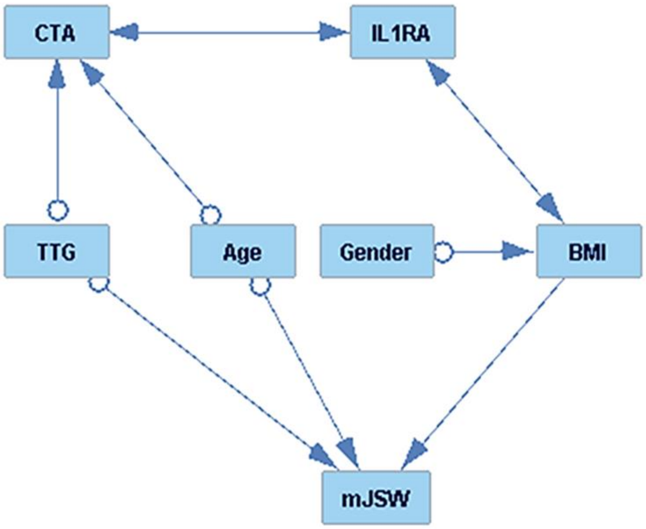


To determine the interdependence of *IL1RN* haplotype, plasma IL-1Ra biomarker, and covariates [body mass index (BMI), age, sex], on medial joint space width (mJSW). FCI algorithm used for causal graph analysis of all variables. Edges with a single arrow denote causality, edges with double arrows denote hidden confounders, and marks (circles) on the edges denote uncertainty of causal orientation. Haplotypes TTG or CTA represents carriers of either *IL1RN* haplotype produced using 3 *IL1RN* SNPs (rs419598, rs315952 and rs9005).
